# Gapless Genome Assembly of Puccinia triticina Provides Insights into Chromosome Evolution in Pucciniales

**DOI:** 10.1128/spectrum.02828-22

**Published:** 2023-01-23

**Authors:** Chuang Li, Liuhui Qiao, Yanan Lu, Guozhen Xing, Xiaodong Wang, Gengyun Zhang, Huimin Qian, Yilin Shen, Yibo Zhang, Wen Yao, Kun Cheng, Zhenling Ma, Na Liu, Daowen Wang, Wenming Zheng

**Affiliations:** a State Key Laboratory of Wheat and Maize Crop Science, College of Life Sciences, Henan Agricultural University, Zhengzhou, China; b State Key Laboratory of Wheat and Maize Crop Science, College of Agronomy and Center for Crop Genome Engineering, Henan Agricultural University, Zhengzhou, China; c State Key Laboratory of North China Crop Improvement and Regulation, College of Plant Protection, Hebei Agricultural University, Baoding, China; d BGI-Shenzhen, Shenzhen, China; USDA—San Joaquin Valley Agricultural Sciences Center

**Keywords:** HiFi sequencing, gapless genome, rust fungi, chromosomal rearrangement, evolutionary radiation

## Abstract

Chromosome evolution drives species evolution, speciation, and adaptive radiation. Accurate genome assembly is crucial to understanding chromosome evolution of species, such as dikaryotic fungi. Rust fungi (Pucciniales) in dikaryons represent the largest group of plant pathogens, but the evolutionary process of adaptive radiation in Pucciniales remains poorly understood. Here, we report a gapless genome for the wheat leaf rust fungus Puccinia triticina determined using PacBio high-fidelity (HiFi) sequencing. This gapless assembly contains two sets of chromosomes, showing that one contig represents one chromosome. Comparisons of homologous chromosomes between the phased haplotypes revealed that highly frequent small-scale sequence divergence shapes haplotypic variation. Genome analyses of *Puccinia triticina* along with other rusts revealed that recent transposable element bursts and extensive segmental gene duplications synergistically highlight the evolution of chromosome structures. Comparative analysis of chromosomes indicated that frequent chromosomal rearrangements may act as a major contributor to rapid radiation of Pucciniales. This study presents the first gapless, phased assembly for a dikaryotic rust fungus and provides insights into adaptive evolution and species radiation in Pucciniales.

**IMPORTANCE** Rust fungi (Pucciniales) are the largest group of plant pathogens. Adaptive radiation is a predominant feature in Pucciniales evolution. Chromosome evolution plays an important role in adaptive evolution. Accurate chromosome-scale assembly is required to understand the role of chromosome evolution in Pucciniales. We took advantage of HiFi sequencing to construct a gapless, phased genome for *Puccinia triticina*. Further analyses revealed that the evolution of chromosome structures in rust lineage is shaped by the combination of transposable element bursts and segmental gene duplications. Chromosome comparisons of *Puccinia triticina* and other rusts suggested that frequent chromosomal arrangements may make remarkable contributions to high species diversity of rust fungi. Our results present the first gapless genome for Pucciniales and shed light on the feature of chromosome evolution in Pucciniales.

## INTRODUCTION

Advances in long-read sequencing technologies have accelerated complete assemblies for complex genomes (1, 2). Highly identical repeats are major limitations due to discrimination errors in these genome projects. PacBio high-fidelity (HiFi) long reads with high accuracy of (>99.9%) conduce to the assembly of complex repeat regions in resolving complex genomes (3). Recently, the completion of the first telomere-to-telomere (T2T) human genome (4) and gapless rice genomes (5, 6) has benefited from HiFi sequencing. More genome projects with the goal of T2T and gapless assemblies are ongoing. Noticeably, most of animals, plants, and fungi contain more than one chromosome copy, and thus, accurate phasing of haplotypes is essential to shedding new light on genome organization and evolution. The emergence of more scaffold-level and haplotype-resolved chromosome assemblies has been greatly driven by long-read sequencing, Hi-C sequencing, and improved algorithms (7–9).

The ultimate goal of assembly is a high-quality haplotype-resolved genome without gaps. The closure of gaps in phased assemblies, especially for complex genomes, is still not trivial. Given that high accuracy of HiFi reads is in favor of haplotype phasing, the assembly software hifiasm (10), designed for haplotype-resolved *de novo* assembly using graph-binning algorithm and maintaining the contiguity of each haplotype, is a powerful tool to obtain phased assembly with high accuracy and contiguity. Other methods are also available in different projects (11). Overall, coupled with different strategies, HiFi reads are expected to help achieve gapless and phased assembly or highly contiguous chromosome sequences.

Dikaryotic fungi carry two different haploid nuclei in a cell along with the two chromosome sets contributing to adaptive evolution. Rust fungi (Pucciniales) in dikaryons are the most speciose natural group of plant pathogens, and many members of this group are the causal agents of prevalent diseases in agriculture and ecology (12, 13). In the life cycle of rust fungi, the predominant stage is generally the dikaryotic developmental phase of urediniospores that recurrently infect hosts. Rust fungi have to enhance their genetic variation to generate intraspecies diversity to overcome numerous host resistances. Comparative genomic analyses have unraveled that sexual recombination and somatic exchange are important drivers of genetic diversity (14, 15). High interhaplotype diversity has been shown to contribute to adaptive evolution (16, 17). Although intraspecies diversity in rust fungi has been reviewed extensively (12, 13), the genetic mechanism driving evolutionary radiation and interspecies diversity is poorly understood (13, 18).

Chromosome evolution plays a pivotal role during speciation and adaptive radiation. It is unclear how chromosomes have evolved to promote species radiation of Pucciniales. Recently, the combination of long-read sequencing and Hi-C data has helped achieve chromosome-level and haplotype-phased assemblies for some rust species, although too many gaps exist in the assembled chromosomes (9, 15, 19–23). Puccinia triticina is the most widely distributed wheat rust fungus (24) and is one of the key species used in rust fungal genomics. To dissect chromosome changes for rust lineage, our study aimed to obtain a highly contiguous, phased chromosome assembly for P. triticina and to investigate the role of chromosome evolution in Pucciniales through evolutionary and comparative analyses.

## RESULTS

### Gapless assembly of *Puccinia triticina* isolate.

*Puccinia triticina* isolate Pt15 was from field samples in central China in 2015. Its virulence profiles were tested using a wheat differential set (see Table S1 in the supplemental material). Whole-genome sequencing of Pt15 yielded 19.83-Gb HiFi reads with a mean length of 14.6 kb and 29.42-Gb Illumina reads (Table S2). hifiasm (10) was used for initial phased assembly with ≥10-kb HiFi reads. Read correction was conducted through two rounds of hifiasm correction in this step. Then, the redundant sequences from heterozygous regions and mitochondria for each haplotype were identified and filtered out, producing two highly contiguous haplotype assemblies (Table S3). Using all long reads, the HERA pipeline (25) was used to connect the remaining contigs of the two haplotypes. The resulting assembly contained 18 contigs totaling 122.82 Mb for haplotype A and 18 contigs totaling 121.11 Mb for haplotype B (Table 1). Given the haploid chromosome number of P. triticina (26) and genome synteny between the two haplotype assemblies, our phased assembly was thought to represent 36 pseudochromosomes in dikaryons. The numbers of chromosomes were assigned according to the length in haplotype A, from the longest, as chromosome 1 (chr1A), to the shortest, as chromosome 18 (chr18A), and homologous chromosomes in haplotype B were identified using haplotype synteny (Fig. S1A). Additionally, our phased genome contained 67 telomeric sequences, and 31 chromosomes contained telomeric sequences at each end (Fig. S1B). Genome synteny between our assembly and Pt76 scaffold-level chromosomes validated the chromosomal structure of the assembled contigs (Fig. S2). The chromosomal structure was also confirmed using Hi-C contact maps by mapping Hi-C sequencing reads from the Pt76 and Pt64 genomes (9, 19) to our assembly (Fig. S3).

**TABLE 1:** Summary of the Pt15 genome assembly and annotation

Parameter	Value for:	
	Haplotype A	Haplotype B
Total length (bp)	122,823,596	121,114,073
Contig no.	18	18
Contig *N*_50_ (bp)	7,485,535	7,574,744
GC content (%)	46.63	46.63
Telomere no.	32	35
Repetitive sequences (%)	63.64	63.31
No. of protein-coding genes	12,737	12,692
Mean gene length (bp)	2,130.81	2,031.33
No. of secreted proteins	1,135	1,119

**FIG 1:**
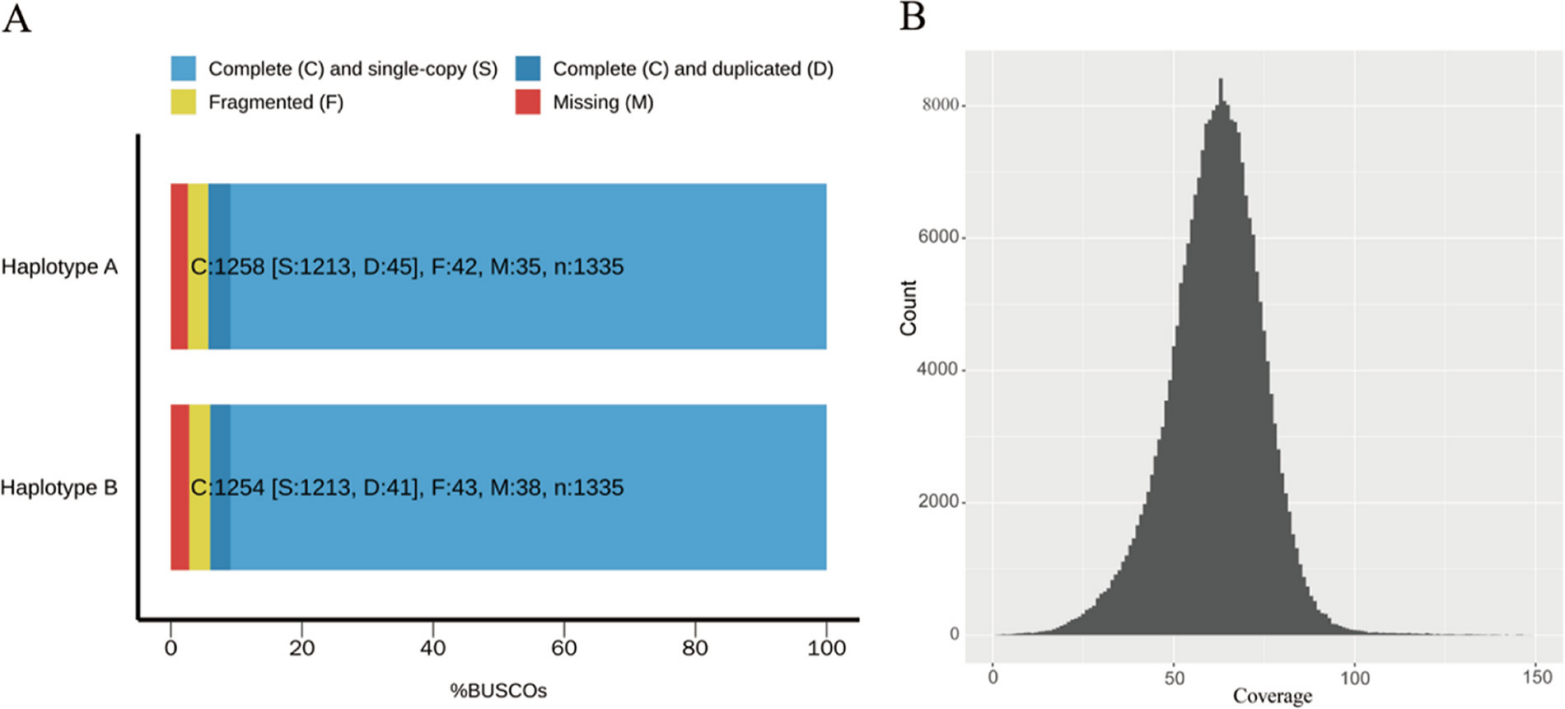
Assessment of genome assembly. (A) Genome completeness of the two haplotypes was assessed using conserved fungal BUSCO genes. (B) Read depth histogram plotted by mapping HiFi reads to the phased assembly, showing full phasing of the assembled genome.

We next evaluated the completeness, continuity, accuracy, and phasing quality of our assembly. Benchmarking Universal Single-Copy Orthologs (BUSCO) (27) analysis for evaluating gene space completeness showed that of 1,335 single-copy orthologues in Basidiomycota, 94.3% and 93.7% were complete in the two haplotypes, respectively. The missing rates of BUSCO genes were 2.6% and 2.8% (Fig. 1A). The long terminal repeat (LTR) assembly index (LAI) was used to assess the continuity of repeat regions (28). The LAI scores of the two haplotypes were 20.63 and 18.93 respectively, which was indicative of a well-assembled genome with many repeats. Long-read coverage analysis at the whole-genome level showed that the two haplotypes were fully phased (Fig. 1B). Merqury analysis (29) with highly accurate Illumina reads showed that the estimated completeness was 97.24% based on k-mers of 19, and the base call accuracy of each chromosome was >QV50 (QV: quality value). Collectively, these analyses demonstrated that our *de novo* phased assembly is of high quality.

We predicted a total of 25,429 protein-coding genes for haplotype A and haplotype B (Table 1; Table S4). BUSCO evaluation showed that the gene annotation completeness was 95.9%, and the missing rate of conserved genes was 1% (Fig. S4). Predicted secreted proteins of the two haplotypes accounted for ~9% of total proteins. Repeat and gene density plots for the chromosomes showed that the distribution of secreted protein genes was not correlated with repeat sequences (Fig. S5).

### Haplotypic variations between homologous chromosomes.

**FIG 2:**
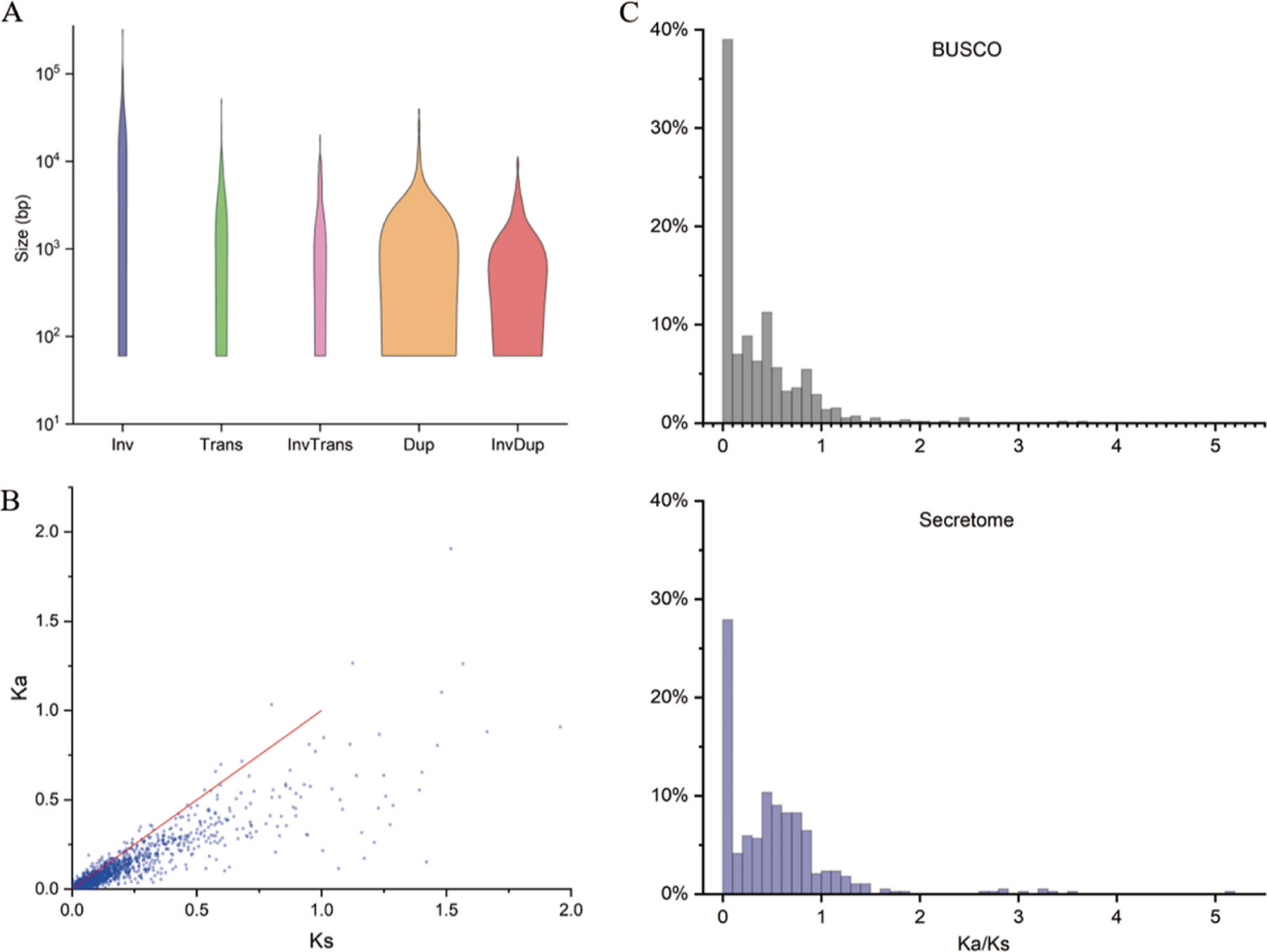
Haplotypic variation. (A) Size distribution of structural variations between homologous chromosomes. Inv, inversions; Trans, translocations; InvTrans, invert translocations; Dup, duplications; InvDup, invert duplications. (B) *K_a_* and *K_s_* distribution for allelic genes. (C) Distribution of *K_a_*/*K_s_* ratios in conserved fungal BUSCO genes and secreted protein genes.

The fully phased genome at the chromosomal level allowed us to discover genomic changes between homologous chromosomes. We found 98.79% sequence identity between the two haplotypes by calculating the average nucleotide identity (ANI) (30). We used Syri (31) to identify structural variations (SVs) between homologous chromosomes (Fig. S6). A total of 108.60 Mb (88.42%) in haplotype A and 108.48 Mb (89.57%) in haplotype B were syntenic blocks. We identified 53 inversions (1,349,548 bp), 73 translocations (280,613 bp), 76 inverted translocations (236,774 bp), 495 duplications (1,544,911 bp), and 370 inverted duplications (587,203 bp) (Table S5). These SVs ranged from over 100 bp to hundreds of kilobases, and the predominant SVs were inversions (Fig. 2A). Sequence variations included 370,259 single nucleotide polymorphisms (SNPs), 19,177 insertions (317,638 bp), and 19,834 deletions (364,941 bp). These variations between homologous chromosomes indicated that haplotypic divergence in Pt15 was largely caused by highly frequent small-scale sequence divergence.

We identified a total of 9,956 allelic gene pairs using gene synteny between homologous chromosomes. All allelic pairs were one-one syntenic genes. All allelic genes were colinear except that only one inversion was found on chr4. Most allelic genes underwent purifying selection, and 5.4% underwent positive selection (Fig. 2B). We compared the evolutionary differences between secreted protein genes and conserved fungal BUSCO genes. The ratio of the number of substitutions per nonsynonymous site to the number of substitutions per synonymous site (*K_a_*/*K_s_* ratio; median, 0.4381; mean, 0.5314) of secreted protein genes was higher than that of BUSCO genes (median, 0.2307; mean, 0.3626). The proportion of positively selected allelic genes encoding secreted proteins was higher than that of BUSCO genes, and the same was true for the proportion of allelic genes that underwent more relaxation of purifying selection (Fig. 2C), suggesting that secreted protein genes evolved more rapidly and were subjected to stronger selective pressure in response to host adaptation.

### Characterization of repetitive sequences.

**FIG 3:**
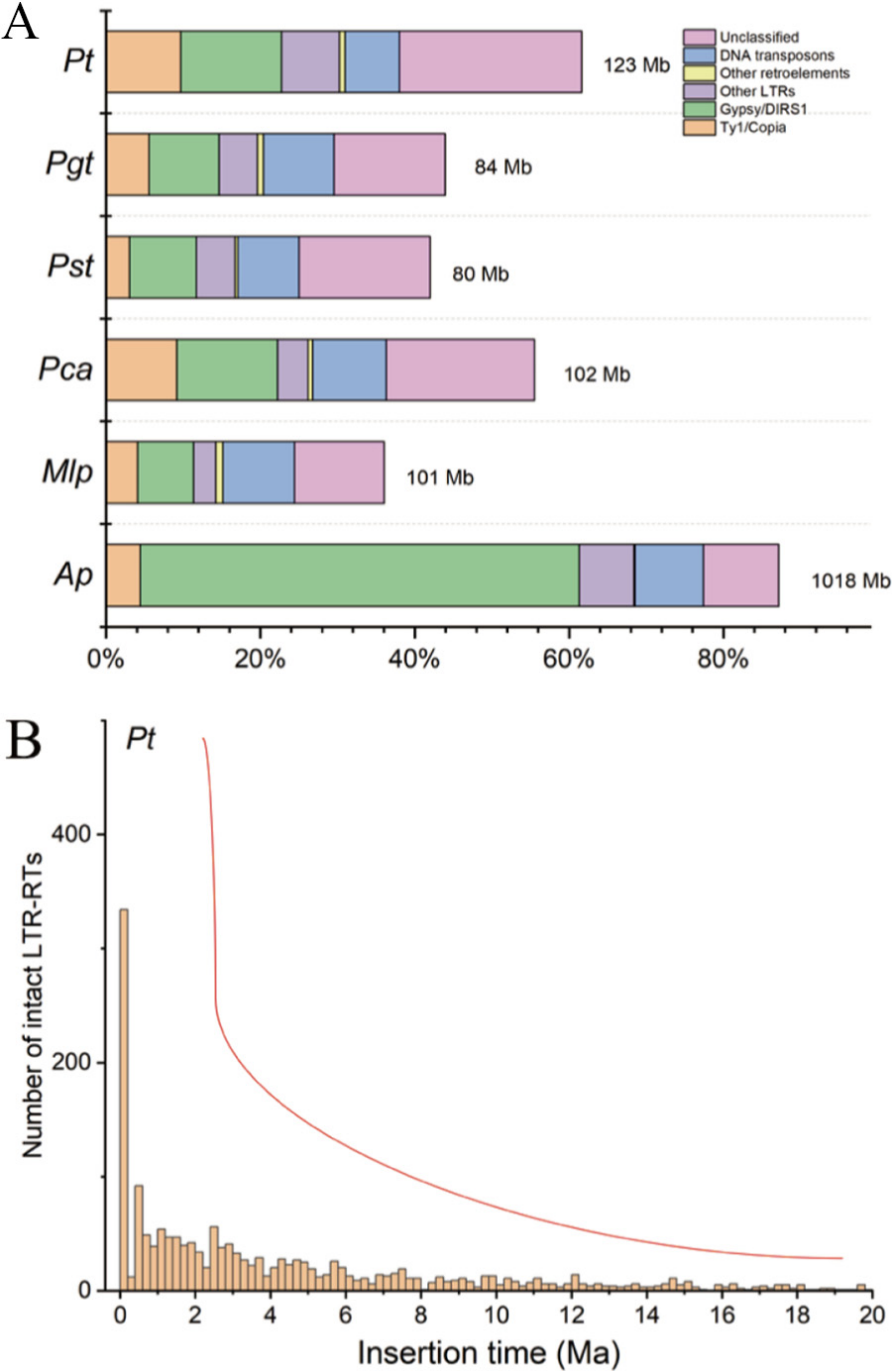
Analysis of TEs in rust species. (A) Comparison of different TEs in P. triticina (*Pt*), P. graminis f. sp. *tritici* (*Pgt*), P. coronata f. sp. *avenae* (*Pca*), P. striiformis f. sp. *tritici* (*Pst*), M. larici-populina (*Mlp*), and A. psidii (*Ap*). (B) Insertion times of LTR-RTs in P. triticina. The red curve represents the trend of the accumulation of TEs in selected rust species. Ma, 1 million years.

The two haplotypes in Pt15 contained similar levels of repetitive sequences (haplotype A, 63.64%; haplotype B, 63.31%). The repetitive sequences were almost evenly distributed on each chromosome (Table S3). The predominant transposable element (TE) type was LTR retrotransposons (LTR-RTs), which mainly included Gypsy and Copia elements (Fig. 3A). We also reanalyzed repetitive sequences of the scaffold-level chromosome assembly for Puccinia graminis f. sp. *tritici*, Puccinia striiformis f. sp. *tritici*, Puccinia coronata f. sp. *avenae*, and Melampsora larici-populina and the primary assembly for Austropuccinia psidii (Table S6). The distribution of TEs in these rust species was similar to that in our assembly (Fig. 3A), indicating that the LTR-RTs, including Gypsy and Copia, and unclassified TEs were the dominant repetitive elements in rust fungi.

We calculated the insertion time of the LTR-RTs in the six genomes (Fig. 3B; Fig. S7), based on the divergence between the LTRs at the ends of each LTR-RT. Although the sequence accuracy of TEs may vary in these genomes and could influence the calculation, the six rust species were found to undergo the significant accumulation of LTR-RTs for genome expansion millions of years ago. Recent large-scale bursts of LTR-RTs were observed over the last 0.2 million years, which contributed dramatically to genome expansion in *Puccinia* species and M. larici-populina. The gigabase-sized genome in A. psidii was attributed to the recent burst and the accumulation over a long period. Given that different TE families in A. psidii showed the burst at discrete time scales (32), we inferred that A. psidii has evolved the complex and efficient mechanism of TE expansion in shaping the supersize of the A. psidii genome compared to those of many species of the rust lineage. Our data also indicated that the expansion of LTR-RTs may still be ongoing.

### Gene duplication drives genome evolution.

**FIG 4:**
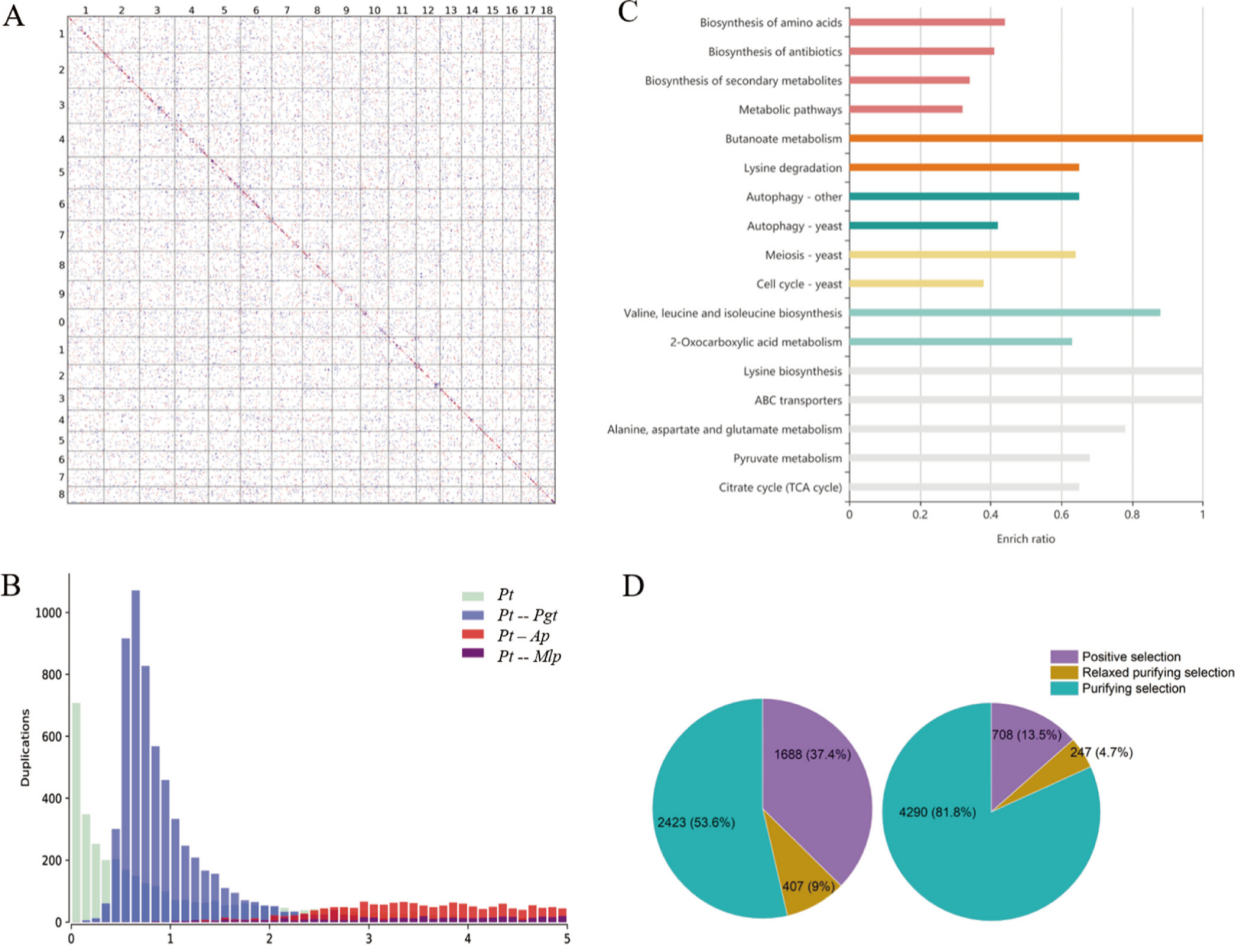
Gene duplication and evolution. (A) Dot plots showing gene duplicates in P. triticina. Chromosome numbers are shown. Best hits, secondary hits, and other hits are shown in red, blue, and gray, respectively. (B) *K_s_* distribution for paralogues in P. triticina and orthologues in P. triticina and other rust species. (C) Bar plot of KEGG pathway enrichment for P. triticina genes after P. triticina-P. graminis divergence. TCA, tricarboxylic acid. (D) Distribution of *K_a_*/*K_s_* ratios for paralogues in P. triticina. (Left) After P. triticina-P. graminis divergence; (right) before P. triticina-P. graminis divergence. Positive selection, relaxed purifying selection, and purifying selection represent *K_a_*/*K_s_* > 1, 0.8 < *K_a_*/*K_s_* < 1, and *K_a_*/*K_s_* ≤ 0.8, respectively.

Gene duplications play a crucial role in species adaptation to diverse environmental changes. Collapsed or incomplete haplotype assembly often leads to errors in the identification of gene duplications in heterozygous diploid genomes. Using whole-genome pairwise alignments of homologous genes in P. triticina, we did not observe significant syntenic blocks within the haploid chromosome set (Fig. 4A) and found that gene duplicates were randomly distributed in the chromosomal structures. Gene collinearity supported the randomness of gene duplicates (Fig. S8). We detected the distribution of *K_s_* values of paralogues in P. triticina, and no *K_s_* peak was observed (Fig. 4B). These findings suggested that P. triticina did not experience recent whole-genome duplications (WGDs) or large-scale gene duplication events, consistent with the observations for *K_s_* distribution of paralogues in P. graminis, P. striiformis f. sp. *tritici*, P. coronata f. sp. *avenae*, A. psidii, and M. larici-populina (Fig. S9). Most gene duplicates in P. triticina and other rust species were dispersed duplicates, which may result from extensive segmental duplications, given that segmental duplication events often occur in eukaryotic genomes with a high TE content (33–35).

We performed Kyoto Encyclopedia of Genes and Genomes (KEGG) pathway analysis of gene duplicates in P. triticina after P. triticina-P. graminis divergence (*K_s_* < 0.7). The results showed that gene duplicates were mainly enriched in pathways including metabolism, biosynthesis, and ABC transporters, which contribute to lifestyle adaptation (Fig. 4C). The number and proportion of paralogues that underwent positive selection (*K_a_*/*K_s_* ratio >1) and relaxed purifying selection (*K_a_*/*K_s_* >0.8) were significantly higher after P. triticina-P. graminis divergence than before P. triticina-P. graminis divergence (Fig. 4D). Species-specific genes in P. triticina were inferred using protein clusters of P. triticina and five other rusts. Species-specific genes shared over one-third of genes that underwent positive selection after P. triticina-P. graminis divergence (Fig. S10), suggesting that rapid evolution of species-specific genes in P. triticina contributed to environmental adaptability.

### Chromosomal rearrangement among rust genomes.

**FIG 5:**
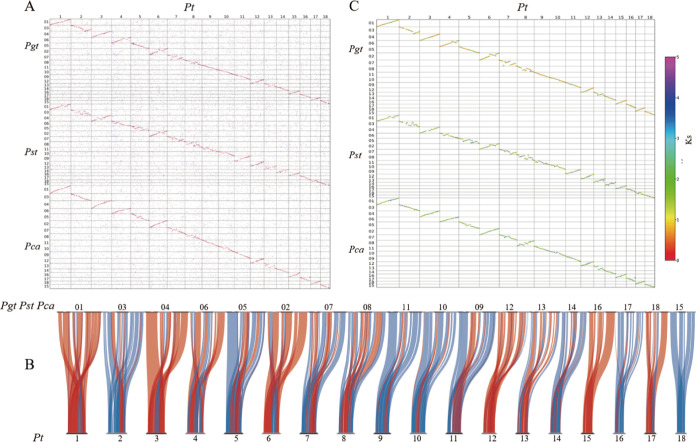
(A) Dot plots showing homologous genes in P. triticina-P. graminis, P. triticina-P. striiformis f. sp. *tritici*, and P. triticina-P. coronata f. sp. *avenae*. Best hits, secondary hits, and other hits are shown in red, blue, and gray, respectively. (B) Gene synteny in P. triticina-P. graminis, P. triticina-P. striiformis f. sp. *tritici*, and P. triticina-P. coronata f. sp. *avenae*. Syntenic blocks containing at least 10 genes are shown. Regular chromosomes are in blue and reversed chromosomes are in red. (C) *K_s_* distribution of gene pairs between syntenic blocks. The median value is shown to represent the *K_s_* distribution of the blocks containing at least 10 gene pairs. Chromosome numbers are shown for the four species.

To investigate chromosome structural changes among rust species, we used P. triticina chromosomes as the reference genome to perform comparative analysis with high-quality genomes of five other rust species. By mapping the P. graminis, P. striiformis f. sp. *tritici*, and P. coronata f. sp. *avenae* chromosome sequences onto the P. triticina genome, we found that overall synteny existed between genomes and genes of these closely related species, and we identified multiple chromosomal rearrangements on different chromosomes, most of which were large inversions (Fig. 5A; Fig. S11). The irregular locations of rearrangements suggested that rearrangement events may occur randomly. In combination with the timescale of TEs of rust genomes in our analysis, the overall genome synteny between P. triticina and the other three *Puccinia* species should be attributed to homologous gene synteny rather than to TEs. Each chromosome of P. graminis, P. striiformis f. sp. *tritici*, and P. coronata f. sp. *avenae* had one matched chromosome in P. triticina (Fig. 5B), meaning that continual rearrangements occurred on each chromosome. These analyses suggested that frequent rearrangements of ancestral chromosomes probably led to the current karyotypes in these species.

P. triticina and P. graminis shared 10,637 syntenic genes of 5,326 pairs that accounted for 34.85% of their total genes. P. triticina and P. striiformis f. sp. *tritici* shared 9,422 syntenic genes of 4,756 pairs that accounted for 33.45% of their total genes. P. triticina and P. coronata f. sp. *avenae* shared 8,407 genes of 4,218 pairs that accounted for 28.58% of their total genes. A total of 5,317, 4,681, and 4,198 genes in P. triticina were syntenic to those of P. graminis, P. striiformis f. sp. *tritici*, and P. coronata f. sp. *avenae*, respectively. We used *K_s_* values of gene pairs between syntenic blocks to evaluate gene divergence among these species. The mean *K_s_* values of P. triticina-P. graminis, P. triticina-P. striiformis f. sp. *tritici*, and P. triticina-P. coronata f. sp. *avenae* were 1.22732, 1.88705, and 1.88731, respectively. These results showed the phylogenetic divergences of the four *Puccinia* species. The *K_s_* comparisons of matched chromosomes of the three species and P. triticina showed that rearrangement may promote gene divergence; for example, gene divergence on chr13 was relatively greater (Fig. 5C; Fig. S12). Altogether, the comparisons suggested that rearrangements may lead to genetic innovations responsible for adaptation and/or speciation.

**FIG 6:**
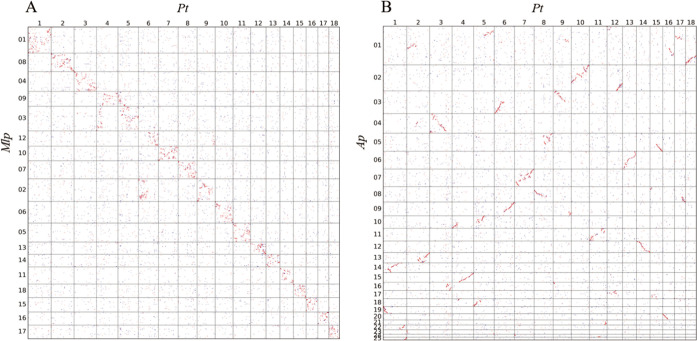
Dot plots showing homologous genes in P. triticina-M. larici-populina (A) and P. triticina-A. psidii (B). Chromosome numbers are shown for P. triticina and M. larici-populina. Sequence numbers are shown for A. psidii.

Subsequently, we detected chromosome structural changes of M. larici-populina and A. psidii from Melampsoraceae and Sphaerophragmiaceae, respectively, compared to P. triticina. For the M. larici-populina chromosome assembly, homologous genes were disordered compared to P. triticina chromosomes but were mostly clustered on each matched chromosome (Fig. 6A). Gene synteny identified retained signals of recurrent chromosome shuffling between M. larici-populina and P. triticina (Fig. S13A). No significant large rearrangement was observed, largely due to the larger phylogenetic distance between M. larici-populina and P. triticina. A high degree of gene order divergence may be attributed to recurrent rearrangements from their ancestral chromosomes. When the supersized assembly in A. psidii was compared to the P. triticina chromosomes, multiple chromosomal rearrangements were detected regardless of the effect of large numbers of TEs (Fig. 6B; Fig. S13B). This is likely due to the phylogenetic relationship between Sphaerophragmiaceae and Pucciniaceae. Chromosome structural changes in M. larici-populina and A. psidii suggested that rearrangements may exist extensively in a broad array of species in the rust lineage.

## DISCUSSION

An accurate, chromosome-level phased genome is necessary to understand the biology and evolution of species, such as dikaryotic fungi. Previous genome projects have shown that Hi-C data appeared to be indispensable for the completion of chromosome-level and phased assembly in dikaryons as well as diploid organisms (15, 19–23). Using higher coverage (80×) of HiFi reads, we obtained a gapless, phased genome of P. triticina isolate Pt15 with hifiasm (10) and HERA (25), and its assembly quality was confirmed by high contiguity and accuracy. While Hi-C sequencing was unavailable in the current project, which limited the assessment of phase switches in the two haplotypes (9), the chromosomal structure and its quality in Pt15 were determined by multiple approaches, such as the assessment of genome data of other P. triticina isolates (9, 19) and the coverage of long reads in the two sets of chromosomes. Overall, this chromosome assembly presents the first gapless, phased genome for a dikaryotic rust fungus and provides a feasible strategy for resolving phased, chromosome-scale genomes of dikaryotic fungi.

The gene set identified in the two haplotypes of Pt15 showed high completeness of conserved fungal genes. Nevertheless, the number of identified genes in each haplotype (12,737 and 12,692) was relatively lower than the previous haplotypes of other P. triticina isolates and *Puccinia* species (9, 21, 22). This was likely due to the effect of Pt15 haplotypes with high contiguity and lack of reductant heterozygous sequences or was associated with the differences in the transcript data.

The dramatic expansions of TEs and gene families have shaped adaptive evolution of rust fungi (13, 36). We evaluated the role of the expansions at the chromosomal scale. Recent bursts of TEs and extensive segmental gene duplications characterize the evolution of chromosome structures in P. triticina and five other rust species, suggesting that TEs and segmental duplications synergistically contribute to genomic adaptation in the rust lineage.

Via comparative analysis of P. triticina chromosomes, we uncovered frequent chromosomal rearrangements in species from Pucciniaceae, Sphaerophragmiaceae, and Melampsoraceae. Given that these species have the larger phylogenetic distance across the rust lineage (13, 37) and lack evidence of recent WGDs, we speculate that chromosome structural changes act regularly and chromosome number changes may be rare in the formation and evolution of modern rust species. This leads to our hypothesis that haploid chromosome number (*n* = 18) may be highly conserved in a wide range of species of the rust lineage, although multiple assembled chromosomes (9, 15, 19–23, 38) support a consistent chromosome number and cytological evidence of karyotypes still remains insufficient (26).

It has been proposed that speciation is driven by chromosomal rearrangements in eukaryotes. For instance, chromosomal inversion can suppress recombination, resulting in reproductive isolation to speciation (39–41). DNA double-strand break repair during the proliferation of TEs has been known to mediate rearrangements (41–43). Strikingly, a relatively higher proportion of TEs in the rust lineage than in other groups of plant pathogens (44–47) may confer the benefit of frequent chromosomal rearrangements, facilitating rapid species radiation by triggering a high rate of formation of new species. This inference is consistent with the observation that species radiation is driven by frequent rearrangements across other eukaryotes, such as the fungal phylum Ascomycota (48) and the angiosperm family Cyperaceae (49). It remains unclear how the occurrence and fixation of chromosomal rearrangements are regulated by TEs or natural selection. Chromosome assembly and comparisons among more diverse taxa are required to reveal the mechanism and trajectory of evolutionary radiation for rust fungi and other fungal lineages.

In summary, this study reports a high-quality gapless genome for P. triticina and provides insights into resolving phased genomes for dikaryotic fungi. Further evolutionary and comparative analyses will enhance the understanding of how chromosome evolution facilitates adaptation and species radiation in Pucciniales.

## MATERIALS AND METHODS

### *Puccinia triticina* isolate and pathotyping.

P. triticina samples were collected from Henan province of China. After single-spore isolation and purification, urediniospores were produced on seedlings of the wheat cultivar Mingxian16. Pathotype identification was tested on the standard wheat differential set for wheat leaf rust.

### DNA extraction and sequencing.

High-molecular-weight DNA was extracted from urediniospores as previously described (50). Qualified DNA was used for constructing a PCR-free SMRT bell library (15 kb) and was sequenced in a SMRT cell on the PacBio Sequel II platform. A PCR-free library for Illumina sequencing was prepared, and 150-bp paired-end reads were produced on the Illumina NovaSeq 6000 platform.

### Genome assembly and evaluation.

Genome assembly was first obtained with HiFi reads using hifiasm 0.15 (10). A BLASTN (51) search against the P. triticina mitochondrial sequences and the NCBI NT database was performed to discard contaminated sequences (52). Heterozygous sequences in each haplotype assembly were identified and removed using Purge Haplotigs (53). HERA (25) was used to construct the final assembly. Hi-C reads for Pt76 and Pt64 were downloaded from the NCBI SRA database (Pt76, SRR14386306; Pt64, SRR14470629). Hi-C contact maps were produced with HiC-Pro 3.10 (54) and HiCExplorer 3.7.1 (55).

Telomeres were identified by searching multiple repeats of TTAGGG or CCCTAA within 200 bp. Genome completeness was assessed using BUSCO 3.02 with genome mode (27) based on the data set basidiomycota_odb9 (2016-02-13). The LAI program (28) was used to evaluate LTRs with default parameters. Genome completeness and accuracy were measured using Merqury (29) with Illumina reads. A genome-wide coverage plot was generated by mapping long reads to genome assembly with the CollapsedGenomicRegions tool (https://github.com/JanaSperschneider/GenomeAssemblyTools).

### Genome annotation.

*De novo* repeats were identified with RepeatModeler 2.0.2a and the option -LTRStruct (56). These repetitive elements and Pucciniomycetes elements from the RepeatMasker library (http://www.repeatmasker.org) (57) were merged as the annotation library, and then RepeatMasker 4.1.2 was run to annotate repetitive sequences. Annotation of protein-coding genes was performed on the repeat-masked genome with the Funannotate pipeline (https://github.com/nextgenusfs/funannotate). Transcript and protein evidence included full-length cDNA from germinated urediniospores in our genome project and transcript sequences from *Puccinia* genomes (Table S6). *Ab initio* gene prediction tool AUGUSTUS (58) was trained using transcript evidence. GeneMark-ES (59) and GlimmerHMM (60) were used for self-training. Finally, all the above-mentioned gene models were combined to produce the consensus gene sets using EvidenceModeler (61).

Gene annotation was evaluated using BUSCO 3.02 with protein mode (27). Secreted proteins were predicted using a combination of SignalP 5.0 (62) and TMHMM 2.0 (63). Repeat and gene densities for the chromosomes were plotted using karyoploteR (64).

### Analysis of LTR-RTs.

Intact LTR-RTs were identified with LTR_retriever (65) based on the merged results from LTR_FINDER (66) and LTRharvest (67). The nucleotide alignments between intact LTR-RTs were performed by MUSCLE (68). The insertion time (*T*) of LTR-RTs was calculated based on the following formula: *T* = *K*/2*r*, where *K* is the divergence rate between the 5′ and 3′ ends of LTRs and *r* is the fungal substitution rate of 1.05 × 10^−9^ nucleotides per site per year (69). *K* was estimated using R package ape (http://ape-package.ird.fr/) with the K80 model.

### Structure variation detection.

The ANI was calculated using fastANI with “–fragLen 1 Mb” (30). Whole-genome alignments between the two haplotypes was performed using Nucmer of MUMmer 3.23 with the parameters “--maxmatch -c 100 -b 500 -l 50,” followed by delta-filter and show-coords (70). SyRi (31) was used to identify genomic rearrangements.

### Analysis of gene duplications.

*K_s_* distributions of paralogues and orthologues were generated using the wgd pipeline (https://github.com/arzwa/wgd) (71). KEGG pathway enrichment analysis was conducted using KOBAS-i (72) with a *P* value of <0.05. OrthoFinder 2.3.3 (73) was used to infer species-specific genes using Markov cluster algorithm (MCL) clustering.

### Synteny analysis.

A whole-genome pairwise alignment of genome assemblies was generated using d-Genies (74). Homologous genes within and between the genomes were identified with their protein sequences by BLASTP alignments (E value < 1e−5). WGDI (75) was used to construct homologous-gene dot plots. Gene synteny was identified using MCScanX (76) and visualized using SynVisio (https://synvisio.github.io/#/).

### *K_a_*/*K_s_* calculation.

The protein sequences were aligned using MUSCLE (68). Protein alignments were converted into coding sequence alignments using PAL2NAL (77). The number of substitutions per nonsynonymous site (*K_a_*), the number of substitutions per synonymous site (*K_s_*), and *K_a_*/*K_s_* values were estimated with the YN00 model in the PAML program (78). *K_s_* values of >5 in the analysis were excluded due to saturation.

### Data availability.

The genome sequence of Pt15 has been deposited in the NCBI database under BioProject numbers PRJNA848635 and PRJNA848634. All sequencing reads are available in the SRA database under BioProject number PRJNA848635.
