# Supplementary material for: Gapless Genome Assembly of Puccinia triticina Provides Insights into Chromosome Evolution in Pucciniales
Source: Microbiol Spectr. 2023 Jan 23;11(1):e02828-22. doi: 10.1128/spectrum.02828-22 (PMC9927501; doi:10.1128/spectrum.02828-22)
Supplement: Supplemental file 1 — Supplemental material. Download spectrum.02828-22-s0001.pdf, PDF file, 1.5 MB [file spectrum.02828-22-s0001.pdf]

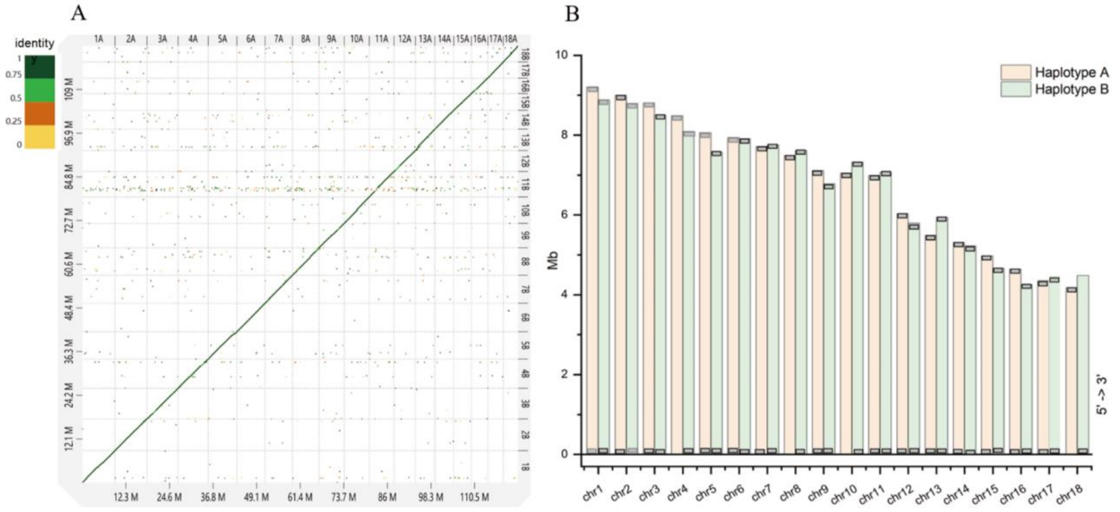

Fig. S1. Chromosome structure in Pt15. **A** Whole-genome pairwise alignment showing homologous chromosome pairs. **B** Chromosome comparison of both haplotypes. Horizontal rectangle indicates telomeric sequence of each chromosome.

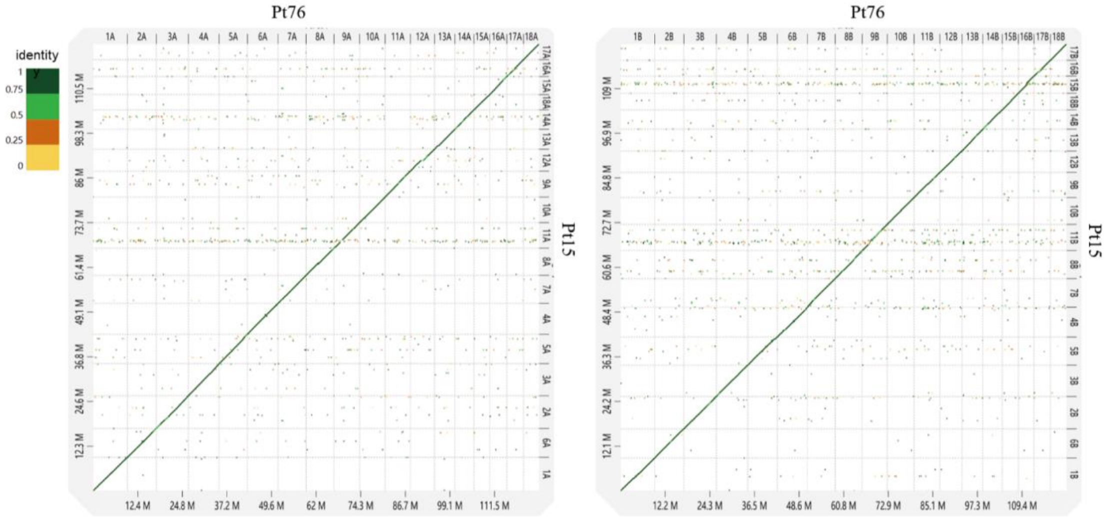

Fig. S2. Whole-genome pairwise alignment of genome assemblies for Pt76 and Pt15.

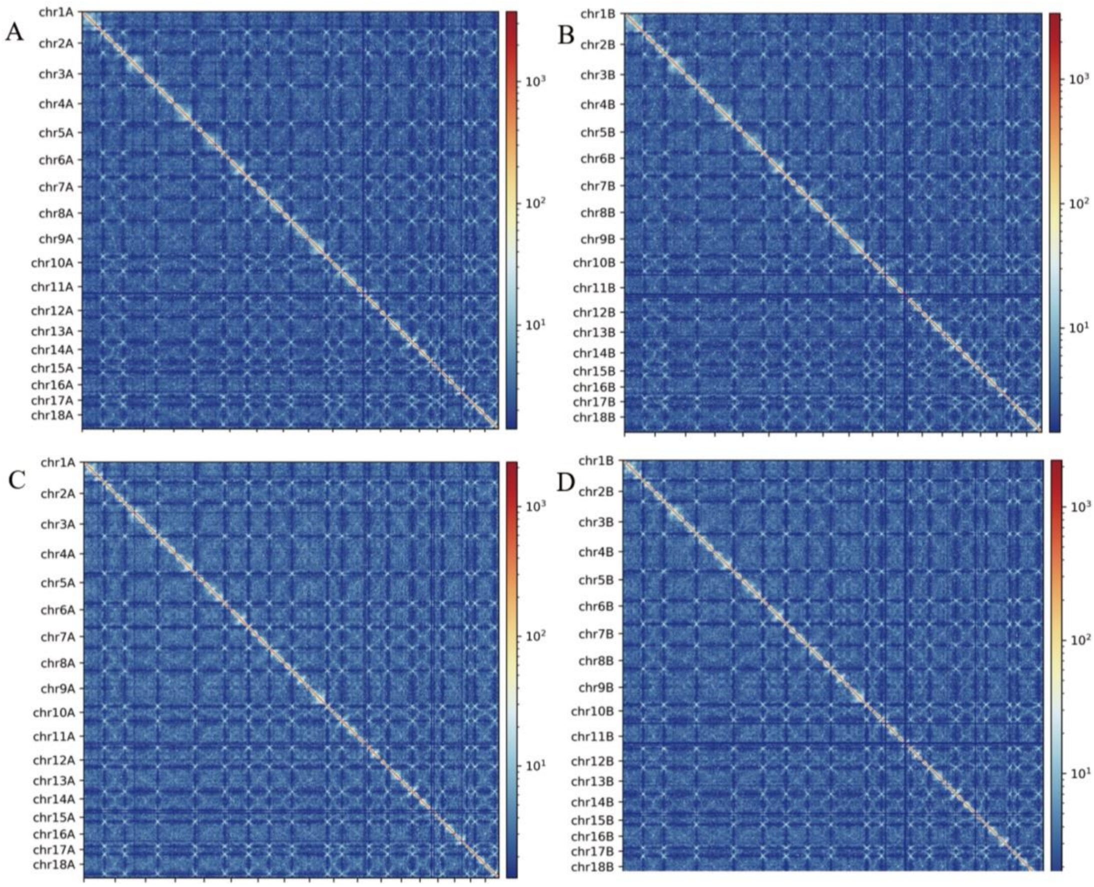

Fig. S3. Hi-C contact maps of genome assembly in Pt15. Hi-C sequencing reads of **(A)** (**B**) and (**C**) (**D**) were from the Pt76 and Pt64 genomes, respectively.

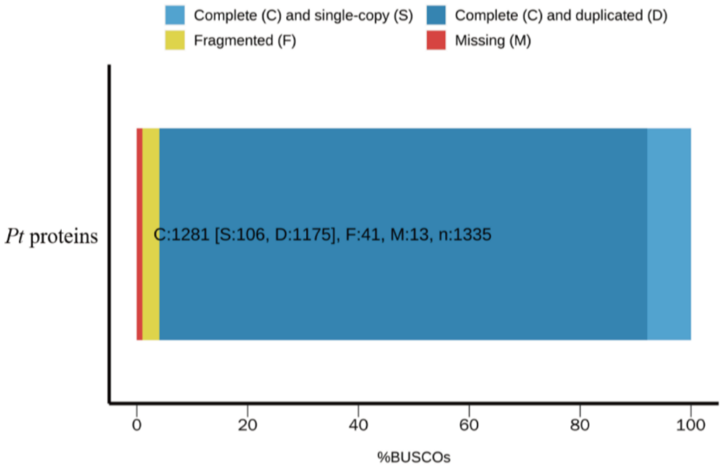

Fig. S4. BUSCO assessment of predicted proteins in Pt15.

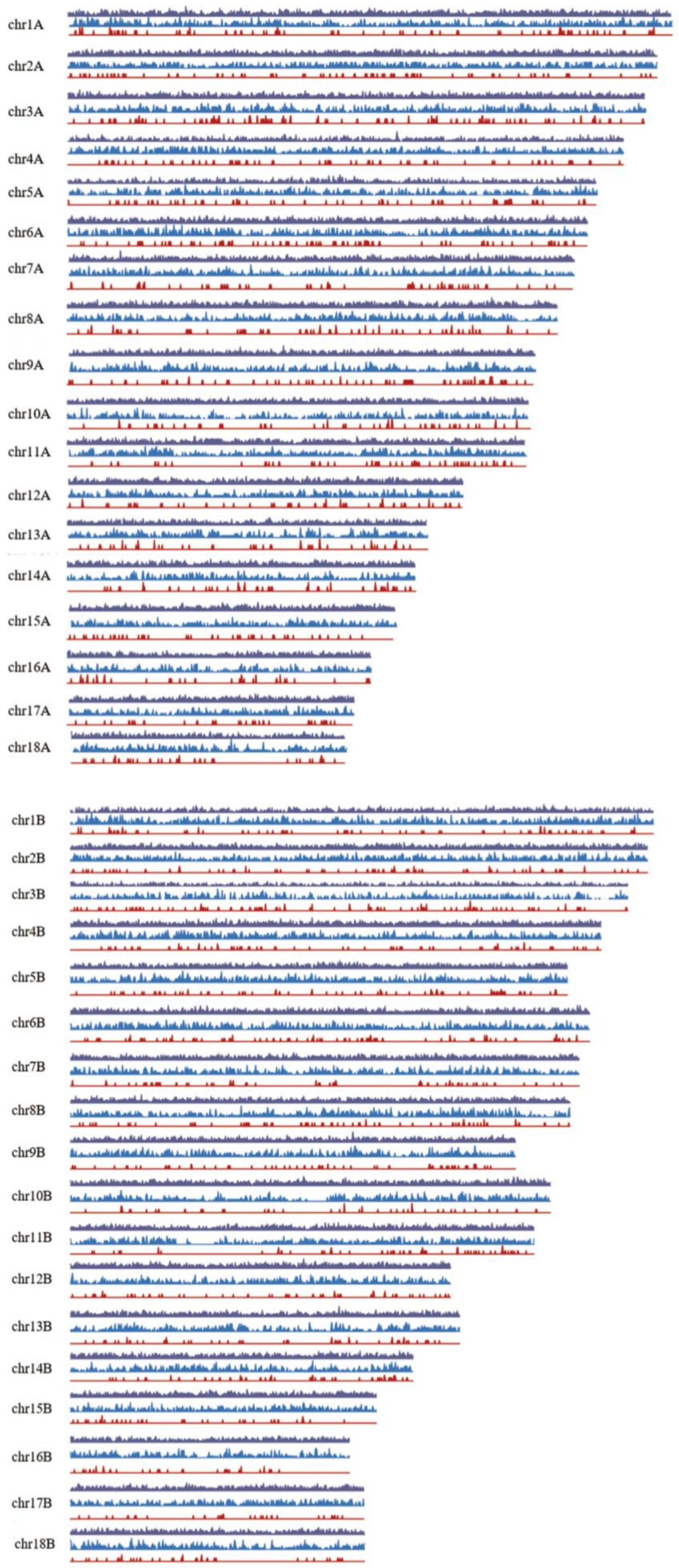

Fig. S5. Repeat and gene densities for the Pt15 chromosomes. From top to bottom, repetitive sequences, all the genes and secreted protein genes are shown for each chromosome, respectively.

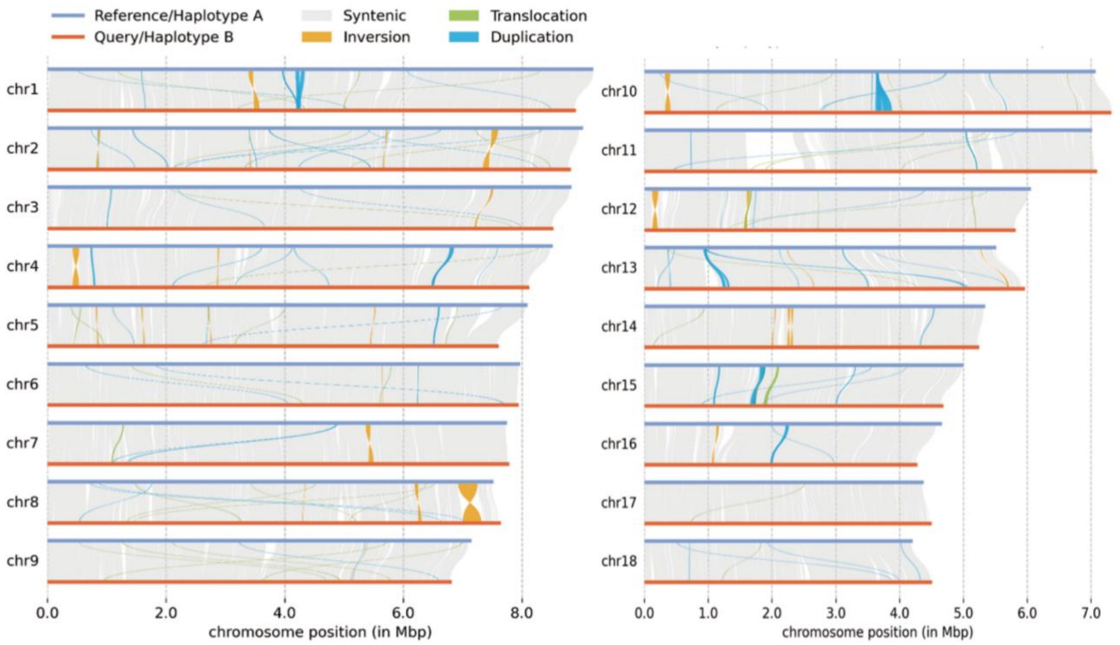

Fig. S6. Structural variation between homologous chromosomes. The blocks greater than 5 kb are shown.

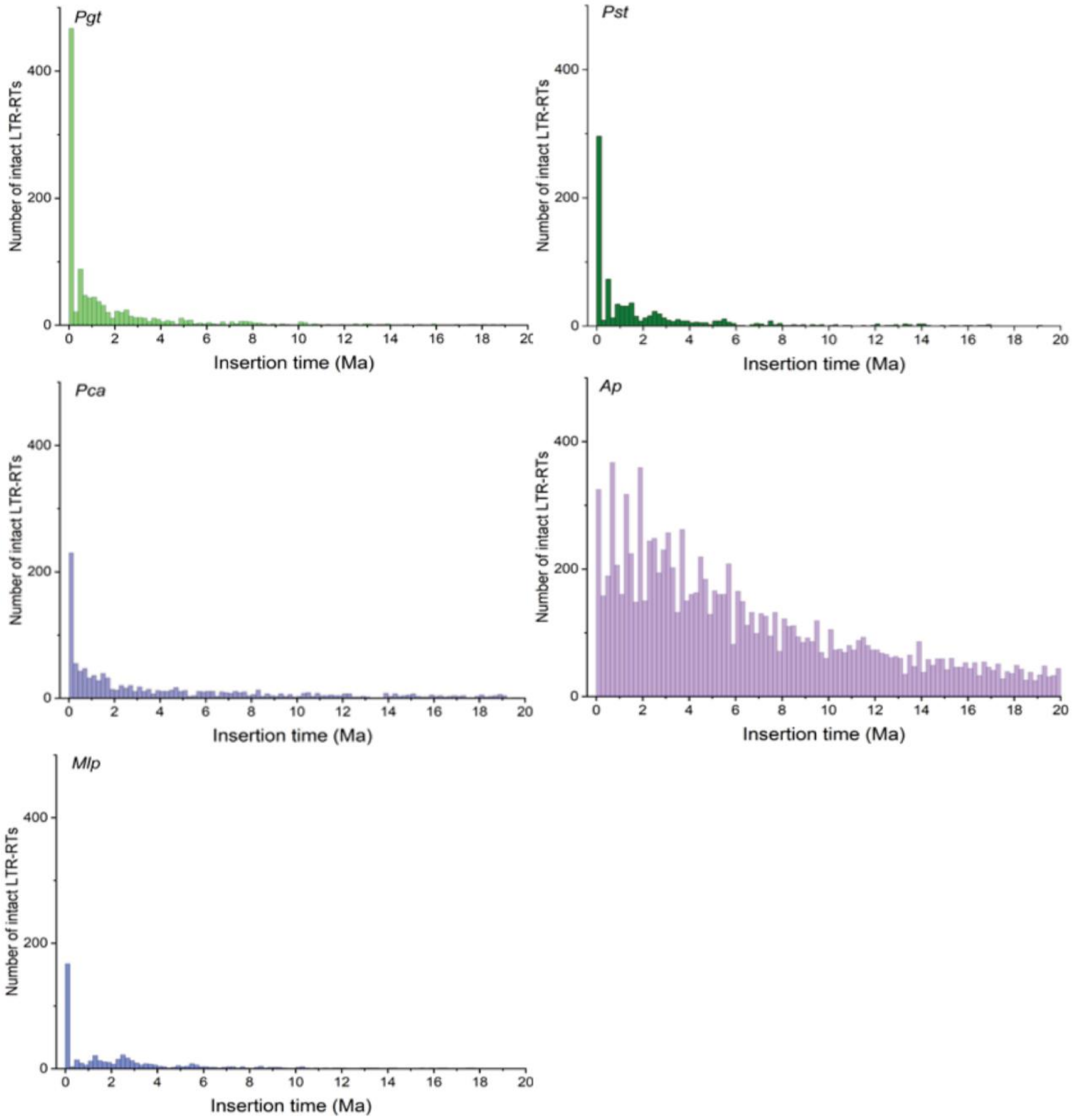

Fig. S7. Insertion time of LTR-RTs in *Pgt*, *Pst*, *Pca*, *Ap* and *Mlp*. MA: one million years.

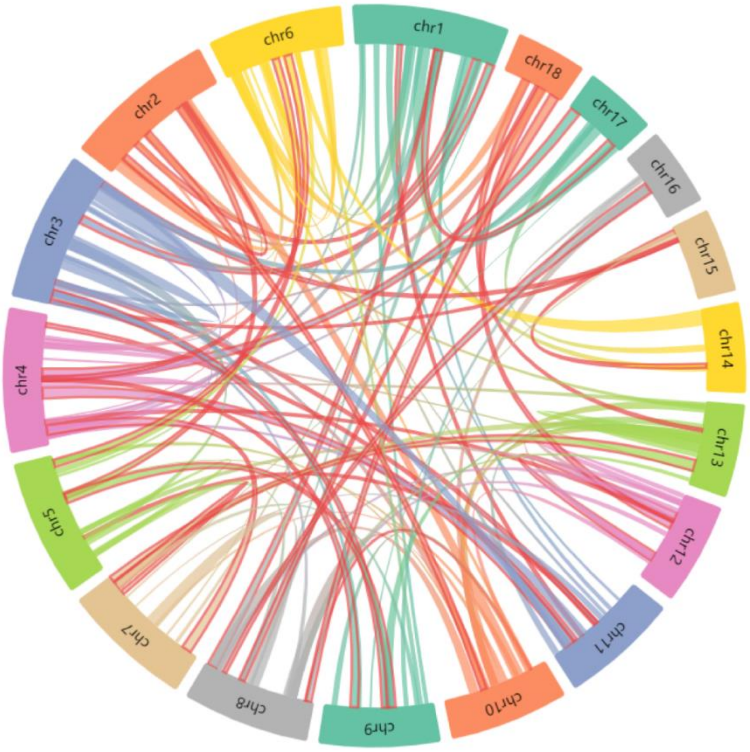

Fig. S8. Gene collinearity showing gene duplicates in *Pt*. MCScanX result is visualized using AccuSyn (https://accusyn.usask.ca/).

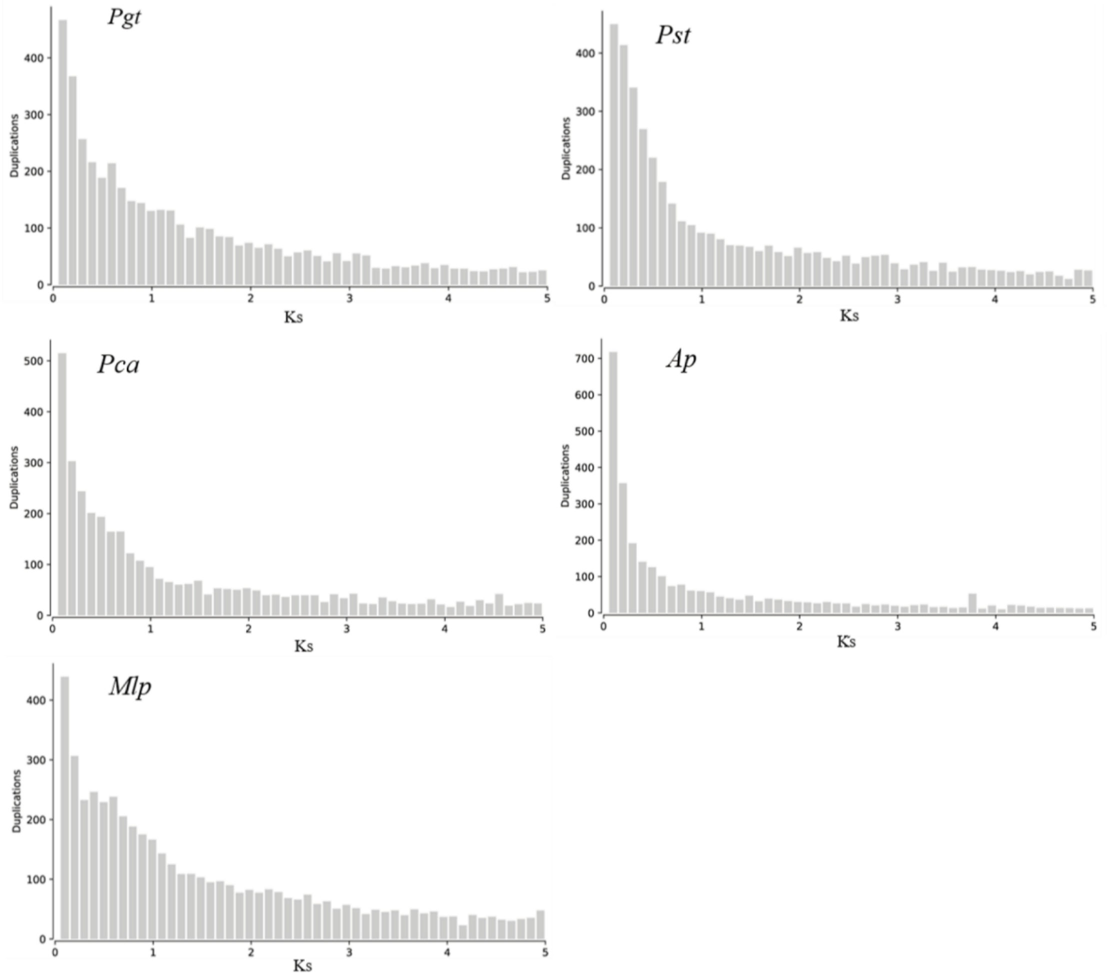

Fig. S9. Ks distribution of paralogues in *Pgt*, *Pst*, *Pca*, *Ap* and *Mlp*.

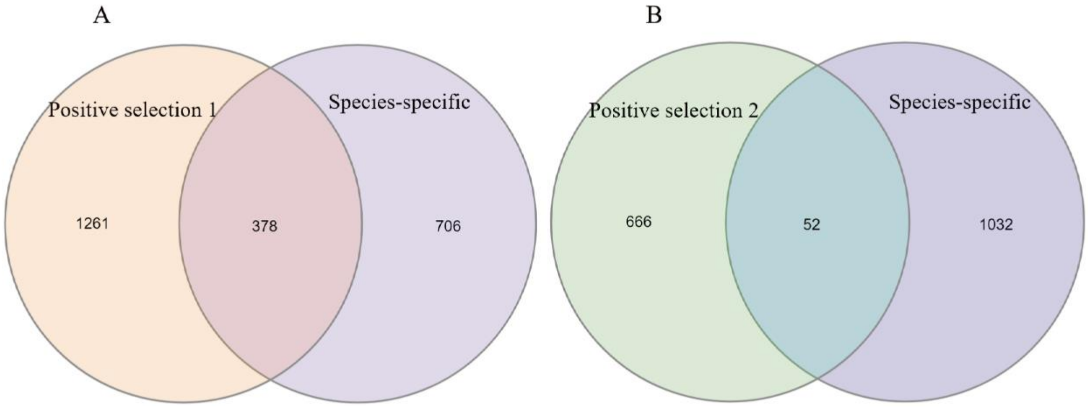

Fig. S10. Venn diagram showing the relation of species-specific genes and genes that underwent positive selection in *Pt*. **A** After *Pt-Pgt* divergence. **B** Before *Pt-Pg*t divergence.

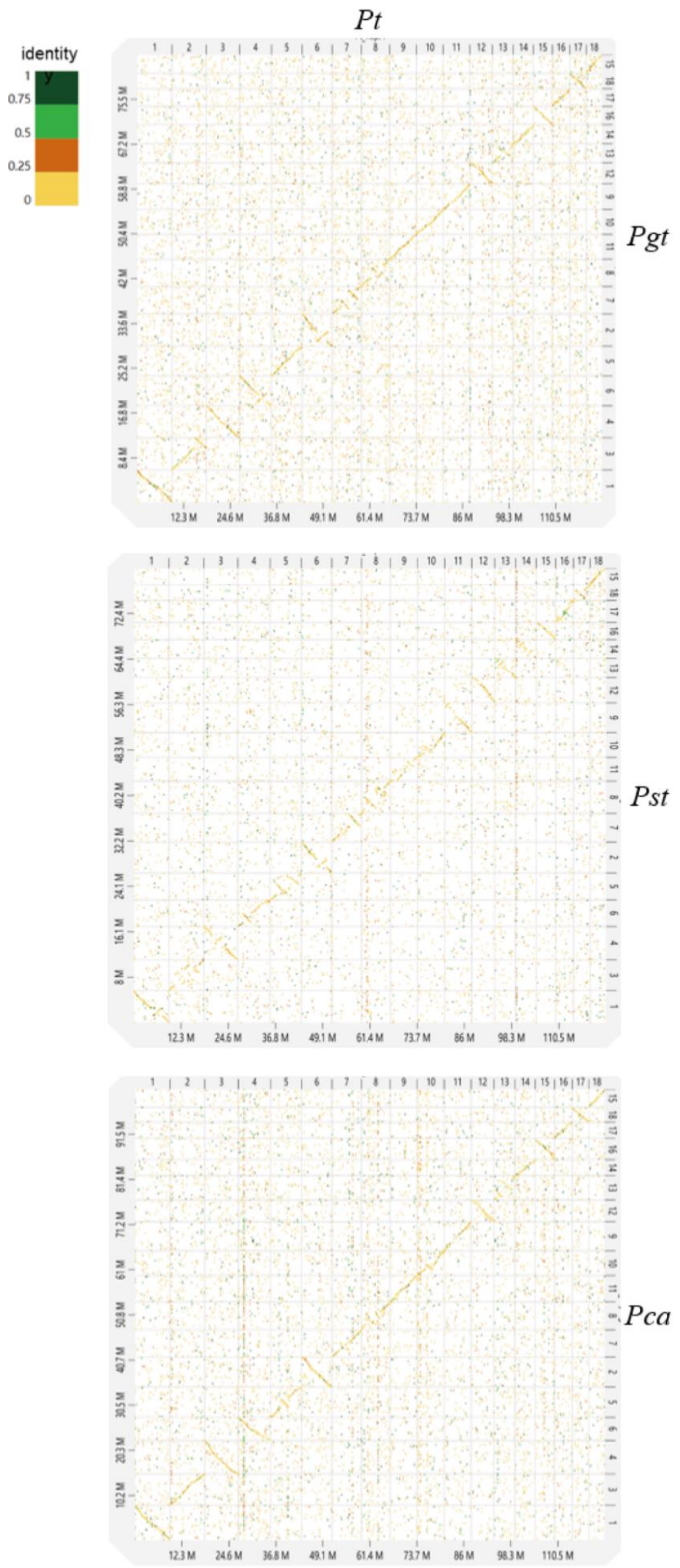

Fig. S11. Whole-genome pairwise alignments between chromosome-level assemblies of *Pt*, *Pgt*, *Pst* and *Pca*. X-axis represents *Pt*. Y-axis represents *Pgt*, *Pst* and *Pca*, respectively.

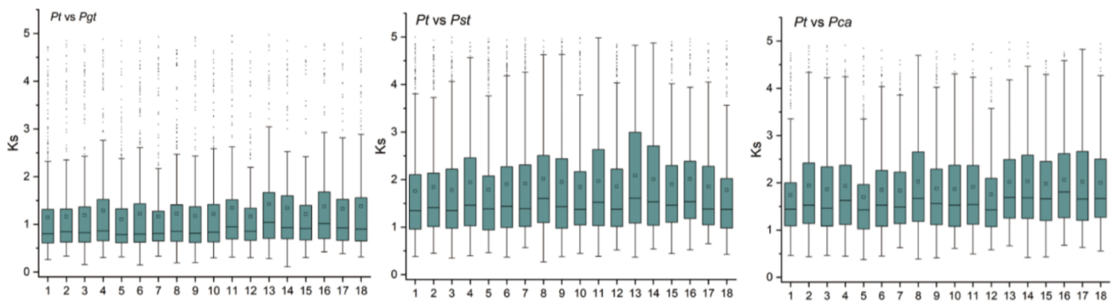

Fig. S12. Ks distribution of gene pairs between syntenic blocks on each chromosome of *Pgt*, *Pst* and *Pca* compared to *Pt*. Chromosome numbers are shown on x-axis.

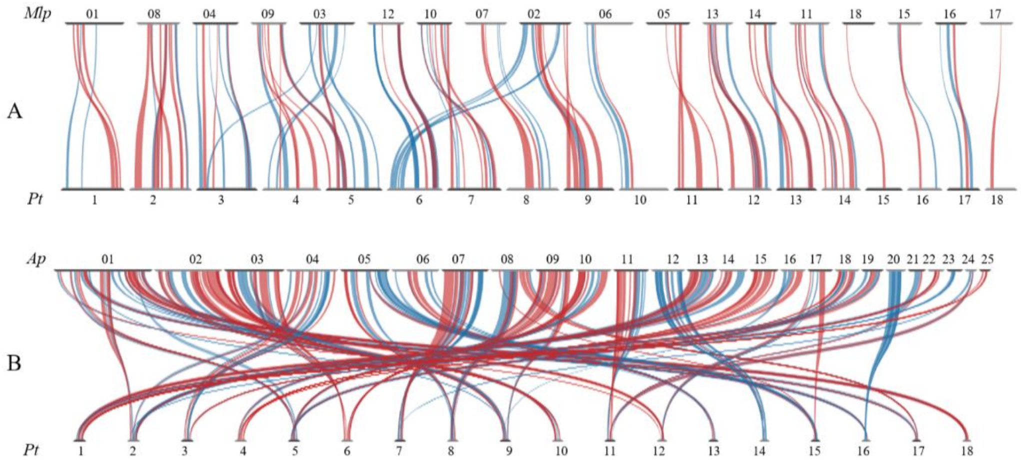

Fig. S13. Gene synteny in *Pt*-*Mlp* (**A**) and *Pt*-*Ap* (**B**). Syntenic blocks containing at least five genes are shown. Chromosome numbers are shown for *Pt* and *Mlp*. Sequence numbers are shown for *Ap*.
